# The PInSoRo dataset: Supporting the data-driven study of child-child and child-robot social dynamics

**DOI:** 10.1371/journal.pone.0205999

**Published:** 2018-10-19

**Authors:** Séverin Lemaignan, Charlotte E. R. Edmunds, Emmanuel Senft, Tony Belpaeme

**Affiliations:** 1 Bristol Robotics Lab, University of the West of England, Bristol, United Kingdom; 2 Centre for Robotics and Neural Systems, University of Plymouth, Plymouth, United Kingdom; 3 IDLab – imec, Ghent University, Ghent, Belgium; University of Texas Medical Branch at Galveston, UNITED STATES

## Abstract

The study of the fine-grained social dynamics between children is a methodological challenge, yet a good understanding of how social interaction between children unfolds is important not only to Developmental and Social Psychology, but recently has become relevant to the neighbouring field of Human-Robot Interaction (HRI). Indeed, child-robot interactions are increasingly being explored in domains which require longer-term interactions, such as healthcare and education. For a robot to behave in an appropriate manner over longer time scales, its behaviours have to be contingent and meaningful to the unfolding relationship. Recognising, interpreting and generating sustained and engaging social behaviours is as such an important—and essentially, open—research question. We believe that the recent progress of machine learning opens new opportunities in terms of both analysis and synthesis of complex social dynamics. To support these approaches, we introduce in this article a novel, open dataset of child social interactions, designed with data-driven research methodologies in mind. Our data acquisition methodology relies on an engaging, methodologically sound, but purposefully underspecified *free-play* interaction. By doing so, we capture a rich set of behavioural patterns occurring in natural social interactions between children. The resulting dataset, called the PInSoRo dataset, comprises 45+ hours of hand-coded recordings of social interactions between 45 child-child pairs and 30 child-robot pairs. In addition to annotations of social constructs, the dataset includes fully calibrated video recordings, 3D recordings of the faces, skeletal informations, full audio recordings, as well as game interactions.

## Introduction

### Studying social interactions

Studying social interactions requires a social *situation* that effectively elicits interactions between the participants. Such a situation is typically scaffolded by a social task, and consequently, the nature of this task influences in fundamental ways the kind of interactions that might be observed and analysed. In particular, the socio-cognitive tasks commonly found in both the experimental psychology and human-robot interaction (HRI) literature often have a narrow focus: because they aim at studying one (or a few) specific social or cognitive skills in isolation and in a controlled manner, these tasks are typically conceptually simple and highly constrained (for instance, object hand-over tasks; perspective-taking tasks; etc.). While these focused endeavours are important and necessary, they do not adequately reflect the complexity and dynamics of real-world, natural interactions (as discussed by Baxter et al. in [1], in the context of HRI). Consequently, we need to investigate richer interactions, scaffolded by socio-cognitive tasks that:

- are long enough and varied enough to elicit a large range of interaction situations;
- foster rich multi-modal interactions, such as simultaneous speech, gesture, and gaze behaviours;
- are not over-specified, in order to maximise natural, non-contrived behaviours;
- evidence complex social dynamics, such as rhythmic coupling, joint attention, implicit turn-taking;
- include a level of non-determinism and unpredictability.

The challenge lies in designing a social task that exhibits these features *while maintaining* essential scientific properties (repeatability; replicability; robust metrics) as well as good practical properties (not requiring unique or otherwise very costly experimental environments; not requiring very specific hardware or robotic platform; easy deployment; short enough experimental sessions to allow for large groups of participants).

Looking specifically at social interactions amongst children, we present in the next section our take on this challenge, and we introduce a novel task of free play. The task is designed to elicit rich, complex, varied social interactions while supporting rigorous scientific methodologies, and is well suited for studying both child-child and child-robot interactions.

### Social play

Our interaction paradigm is based on free and playful interactions (hereafter, *free play*) in what we call a *sandboxed environment*. In other words, while the interaction is free (participants are not directed to perform any particular task beyond playing), the activity is both *scaffolded* and *constrained* by the setup mediating the interaction (a large interactive table), in a similar way to children freely playing with sand within the boundaries of a sandpit. Consequently, while participants engage in open-ended and non-directed activity, the play situation is framed to be easily reproducible as well as practical to record and analyse.

This initial description frames the socio-cognitive interactions that might be observed and studied: playful, dyadic, face-to-face interactions. While gestures and manipulations (including joint manipulations) play an important role in this paradigm, the participants do not typically move much during the interaction. Because it builds on play, this paradigm is also primarily targeted to practitioners in the field of child-child or child-robot social interactions.

The choice of a playful interaction is supported by the wealth of social situations and social behaviours that play elicits (see for instance parts 3 and 4 of [2]). Most of the research in this field builds on the early work of Parten who established five *stages of play* [3], corresponding to different stages of development, and accordingly associated with typical age ranges: (*a*) *solitary (independent) play* (age 2-3): child playing separately from others, with no reference to what others are doing; (*b*) *onlooker play* (age 2.5-3.5): child watching others play; may engage in conversation but not engage in doing; true focus on the children at play; (*c*) *parallel play* (also called adjacent play, social co-action, age 2.5-3.5): children playing with similar objects, clearly beside others but not with them; (*d*) *associative play* (age 3-4): child playing with others without organization of play activity; initiating or responding to interaction with peers; (*e*) *cooperative play* (age 4+): coordinating one’s behavior with that of a peer; everyone has a role, with the emergence of a sense of belonging to a group; beginning of “team work.”

These five stages of play have been extensively discussed and refined over the last century, yet remain remarkably widely accepted. It must be noted that the age ranges are only indicative. In particular, most of the early behaviours still occur at times by older children.

### Machine learning, robots and social behaviours

The data-driven study of social mechanisms is still an emerging field, and only limited literature is available.

The use of interaction datasets to teach artificial agents (robots) how to socially behave has been previously explored, and can be considered as the extension of the traditional learning from demonstration (LfD) paradigms to social interactions [4, 5]. However, existing research focuses on low-level identification or generation of brief, isolated behaviours, including social gestures [6] and gazing behaviours [7].

Based on a human-human interaction dataset, Liu et al. [8] have investigated machine learning approaches to learn longer interaction sequences. Using unsupervised learning, they train a robot to act as a shop-keeper, generating both speech and socially acceptable motions. Their approach remains task-specific, and they report only limited success. They however emphasise the “life-likeness” of the generated behaviours.

This burgeoning interest in the research community for the data-driven study of social responses is however impaired by the lack of structured research efforts. In particular, there is only limited availability of large and open datasets of social interactions, suitable for machine-learning applications.

One such dataset is the *Multimodal Dyadic Behavior Dataset* (*MMDB*, [9]). It comprises of 160 sessions of 3 to 5 minute child-adult interactions. During these interactions, the experimenter plays with toddlers (1.5 to 2.5 years old) in a semi-structured manner. The dataset includes video streams of the faces and the room, audio, physiological data (electrodermal activity) as well as manual annotations of specific behaviours (like gaze to the examiner, laughter, pointing). This dataset focuses on very young children during short, adult-driven interactions. As such, it does not include episodes of naturally-occurring social interactions between peers, and the diversity of said interactions is limited. Besides, the lack of intrinsic and extrinsic camera calibration information in the dataset prevent the automatic extraction and labeling of key interaction features (like mutual gaze).

Another recent dataset, the *Tower Game Dataset* [10], focuses specifically on rich dyadic social interactions. The dataset comprises of 39 adults recorded over a total of 112 annotated sessions of 3 min in average. The participants are instructed to jointly construct a tower using wooden blocks. Interestingly, the participants are not allowed to talk to maximise the amount of non-verbal communication. The skeletons and faces of the participants are recorded, and the dataset is manually annotated with so-called *Essential Social Interaction Predicates* (ESIPs): rhythmic coupling (entrainment or attunement), mimicry (behavioral matching), movement simultaneity, kinematic turn taking patterns, joint attention. This dataset does not appear to be publicly available on-line.

The UE-HRI dataset [11] is another recently published (2017) dataset of social interactions, focusing solely on human-robot interactions. 54 adult participants were recorded (duration M = 7.7min) during spontaneous dialogues with a Pepper robot. The interactions took place in a public space, and include both one-to-one and multi-party interactions. The resulting dataset includes audio and video recordings from the robot perspective, as well as manual annotations of the levels of engagement. It is publicly available.

PInSoRo, our dataset, shares some of the aims of the *Tower Game* and *UE-HRI* datasets, with however significant differences. Contrary to these two datasets, our target population are children. We also put a strong focus on naturally occurring, real-world social behaviours. Furthermore, as presented in the following sections, we record much longer interactions (up to 40 minutes) of free play interactions, capturing a wider range of socio-cognitive behaviours. We did not place any constraints on the permissible communication modalities, and the recordings were manually annotated with a focus on social constructs.

## Material and methods

### The free-play sandbox task

**Fig 1:**
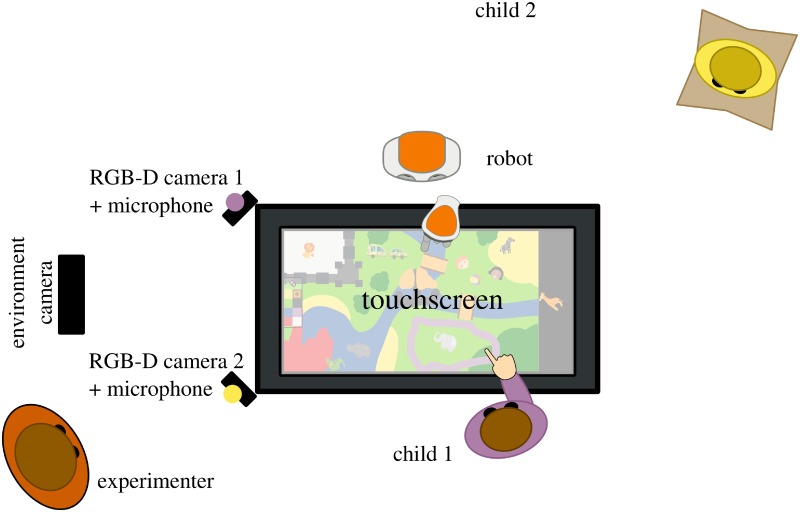
The free-play social interactions sandbox: Two children or one child and one robot (as pictured here) interacted in a free-play situation, by drawing and manipulating items on a touchscreen. Children were facing each other and sit on cushions. Each child wore a bright sports bib, either purple or yellow, to facilitate later identification.

As previously introduced, the *free-play sandbox* task is based on face-to-face free-play interactions, mediated by a large, horizontal touchscreen. Pairs of children (or alternatively, one child and one robot) are invited to freely draw and interact with items displayed on an interactive table, without any explicit goals set by the experimenter (Fig 1). The task is designed so that children can engage in open-ended and non-directive play. Yet, it is sufficiently constrained to be suitable for recording, and allows the reproduction of social behaviour by an artificial agent in comparable conditions.

**Fig 2:**
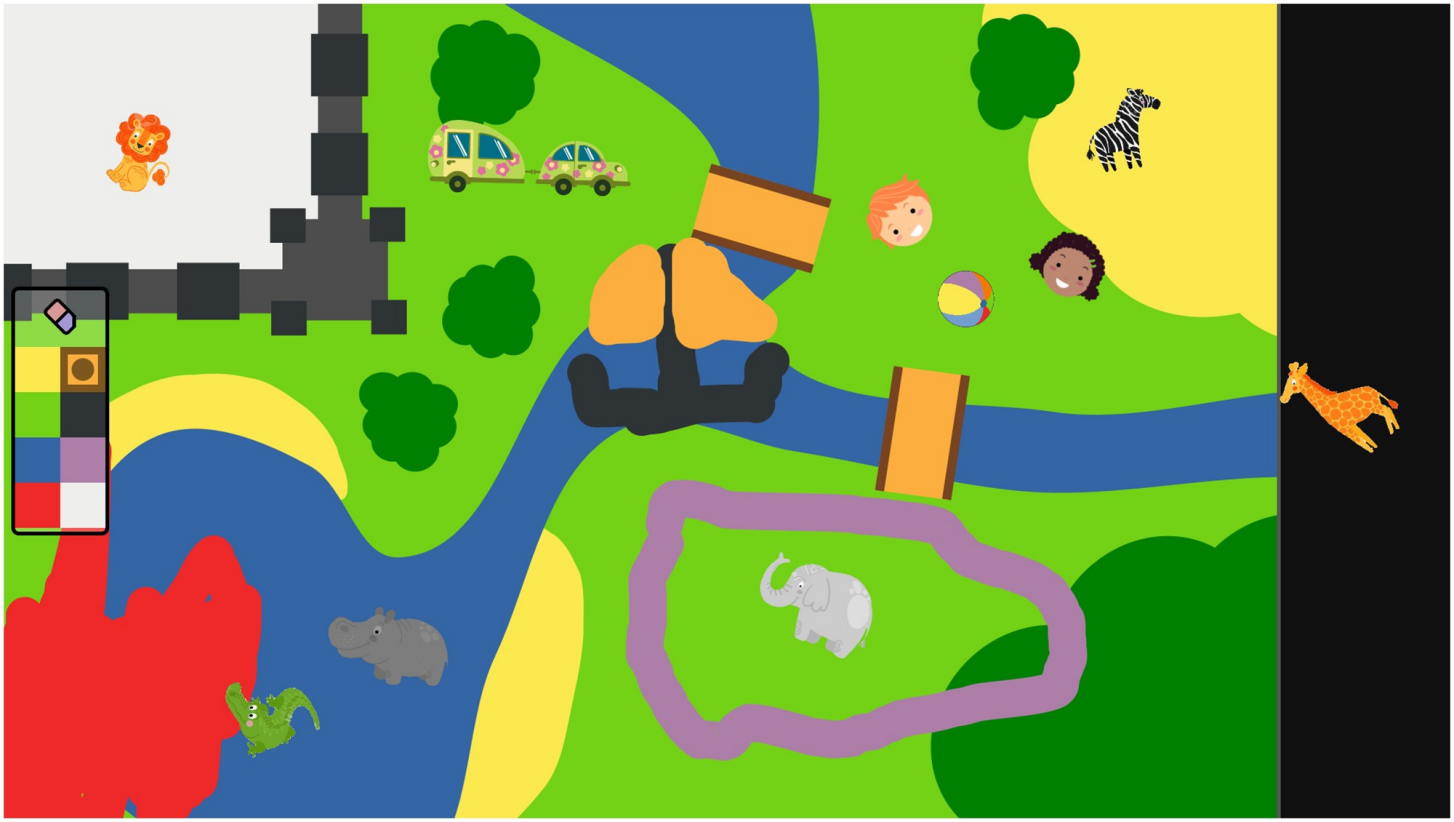
Example of a possible game situation. Game items (animals, characters…) can be dragged over the whole play area, while the background picture can be painted over by picking a colour. In this example, the top player is played by a robot.

Specifically, the free-play sandbox follows the *sandtray* paradigm [12]: a large touchscreen (60cm × 33cm, with multitouch support) is used as an interactive surface. The two players, facing each other, play together, moving interactive items or drawing on the surface if they wish so (Fig 2). The background image depicts a generic empty environment, with different symbolic colours (water, grass, beach, bushes…). By drawing on top of the background picture, the children can change the environment to their liking. The players do not have any particular task to complete, they are simply invited to freely play. They can play for as long as they wish. However, for practical reasons, we had to limit the sessions to a maximum of 40 minutes.

Even though the children do typically move a little, the task is fundamentally a face-to-face, spatially delimited, interaction, and as such simplifies the data collection. In fact, the children’s faces were successfully detected in 98% of the over 2 million frames recorded during the PInSoRo dataset acquisition campaign.

#### Experimental conditions

The PInSoRo dataset aims to establish two experimental baselines for the free-play sandbox task: the ‘human social interactions’ baseline on one hand (child–child condition), an ‘asocial’ baseline on the other hand (child–*non-social* robot condition). These two baselines aim to characterise the qualitative and quantitative bounds of the spectrum of social interactions and dynamics that can be observed in this situation.

In the *child-child* condition, a diverse set of social interactions and social dynamics were expected to be observed, ranging from little social interactions (for instance, with shy children) to strong, positive interactions (for instance, good friends), to hostility (children who do not get along very well).

In the *asocial* condition, one child was replaced by an autonomous robot. The robot was purposefully programmed to be *asocial*. It autonomously played with the game items as a child would (although it did not perform any drawing action), but avoided all social interactions: no social gaze, no verbal interaction, no reaction to child-initiated game actions.

From the perspective of social psychology, this condition provides a baseline for the social interactions and dynamics at play (or the lack thereof) when the social communication channel is severed between the agents, while maintaining a similar social setting (face-to-face interaction; free-play activity).

From the perspective of human-robot interaction and artificial intelligence in general, the child–‘asocial robot’ condition provides a baseline to contrast with for yet-to-be-created richer social and behavioural AI policies.

#### Hardware apparatus

The interactive table was based on a 27” Samsung All-In-One computer (quad core i7-3770T, 8GB RAM) running Ubuntu Linux and equipped with a fast 1TB SSD hard-drive. The computer was held horizontally in a custom aluminium frame standing 26cm above the floor. All the cameras were connected to the computer via USB-3. The computer performed all the data acquisition using ROS Kinetic (http://www.ros.org/). The same computer was also running the game interface on its touch-enabled screen (60cm × 33cm), making the whole system standalone and easy to deploy.

The children’s faces were recorded using two short range (0.2m to 1.2m) Intel RealSense SR300 RGB-D cameras placed at the corners of the touchscreen (Fig 1) and tilted to face the children. The cameras were rigidly mounted on custom 3D-printed brackets. This enabled a precise measurement of their 6D pose relative to the touchscreen (extrinsic calibration).

Audio was recorded from the same SR300 cameras (one mono audio stream was recorded for each child, from the camera facing him or her).

Finally, a third RGB camera (the RGB stream of a Microsoft Kinect One, the *environment camera* in Fig 1) recorded the whole interaction setting. This third video stream was intended to support human coders while annotating the interaction, and was not precisely calibrated.

In the child-robot condition, a Softbank Robotics’ Nao robot was used. The robot remained in standing position during the entire play interaction. The actual starting position of the robot with respect to the interactive table was recalibrated before each session by flashing a 2D fiducial marker on the touchscreen, from which the robot could compute its physical location.

#### Software apparatus

The software-side of the free-play sandbox is entirely open-source (source code: https://github.com/freeplay-sandbox/). It was implemented using two main frameworks: Qt QML (http://doc.qt.io/qt-5/qtquick-index.html) for the user interface (UI) of the game (Fig 2), and the *Robot Operating System* (ROS) for the modular implementation of the data processing and behaviour generation pipelines, as well as for the recordings of the various datastreams (Fig 4). The graphical interface interacts with the decisional pipeline over a bidirectional QML-ROS bridge that was developed for that purpose (source code available from the same link).

**Fig 3:**
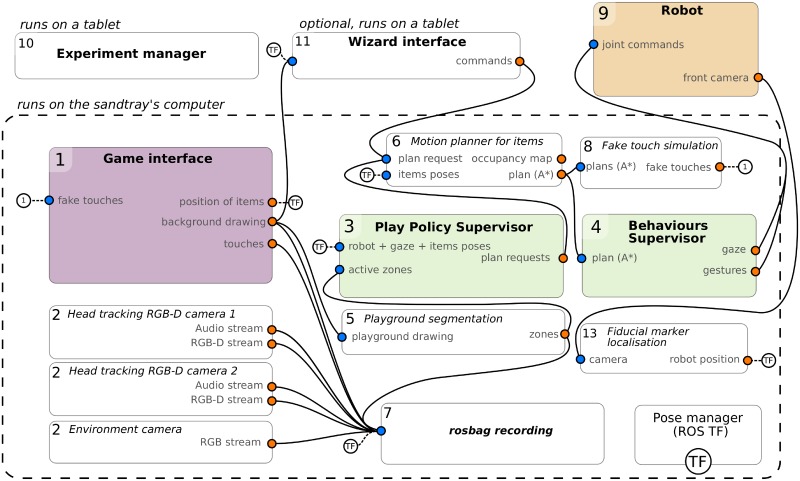
Software architecture of the free-play sandbox (data flows *from* orange dots *to* blue dots). Left nodes interact with the interactive table hardware (game interface (1) and camera drivers (2)). The green nodes in the centre implement the behaviour of the robot (play policy (3) and robot behaviours (4)). Several helper nodes are available to provide for instance a segmentation of the children drawings into zones (5) or A* motion planning for the robot to move in-game items (6). Nodes are implemented in Python (except for the game interface, developed in QML) and inter-process communication relies on ROS. 6D poses are managed and exchanged via ROS TF.

Fig 3 presents the complete software architecture of the sandbox as used in the child-robot condition (in the child-child condition, robot-related modules were simply not started).

#### Robot control

As previously described, one child was replaced by a robot in the child-robot condition. Our software stack allowed for the robot to be used in two modes of operations: either autonomous (selecting actions based on pre-programmed play policies), or controlled by a human operator (so-called *Wizard-of-Oz* mode of operation).

For the purpose of the PInSoRo dataset, the robot behaviour was fully autonomous, yet coded to be purposefully *asocial* (no social gaze, no verbal interaction, no reaction to child-initiated game actions). The simple action policy that we implemented consisted in the robot choosing a random game item (in its reach), and moving that item to a predefined zone on the map (e.g. if the robot could reach the crocodile figure, it would attempt to drag it to a blue, i.e. water, zone). The robot did not physically drag the item on the touchscreen: it relied on a A* motion planner to find an adequate path, sent the resulting path to the touchscreen GUI to animate the displacement of the item, and moved its arm in a synchronized fashion using the inverse kinematics solver provided with the robot’s software development kit (SDK).

**Fig 4:**
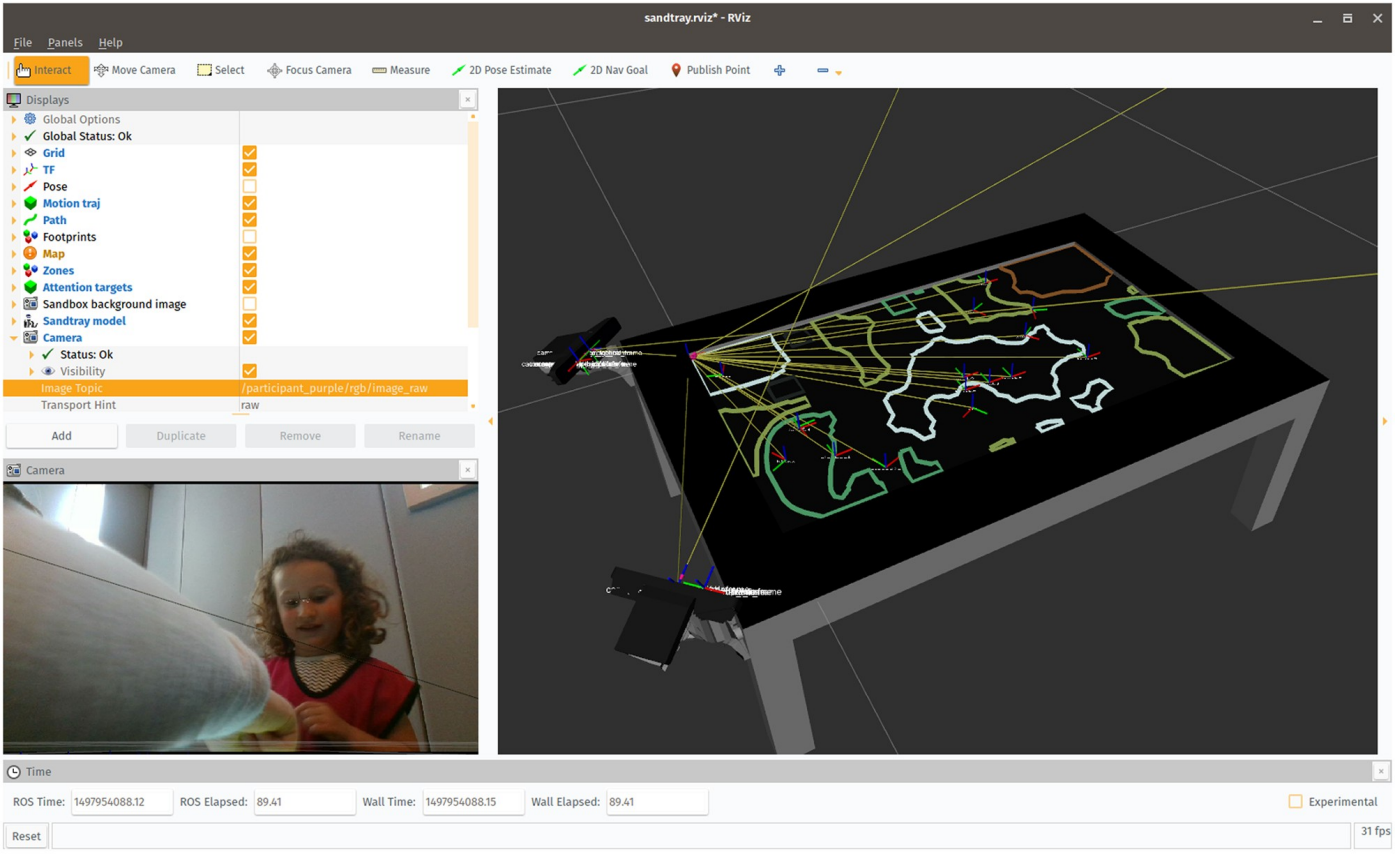
The free-play sandbox, viewed at runtime within ROS RViz. Simple computer vision was used to segment the background drawings into zones (visible on the right panel). The poses and bounding boxes of the interactive items were broadcast as well, and turned into an occupancy map, used to plan the robot’s arm motion. The individual pictured in this figure has given written informed consent (as outlined in PLOS consent form) to appear.

**Fig 5:**
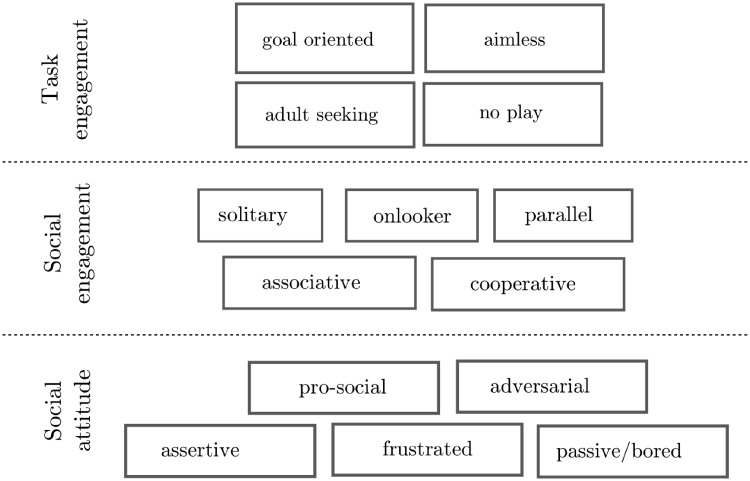
The coding scheme used for annotating social interactions occurring during free-play episodes. Three main axis were studied: task engagement, social engagement and social attitude.

**Fig 6:**
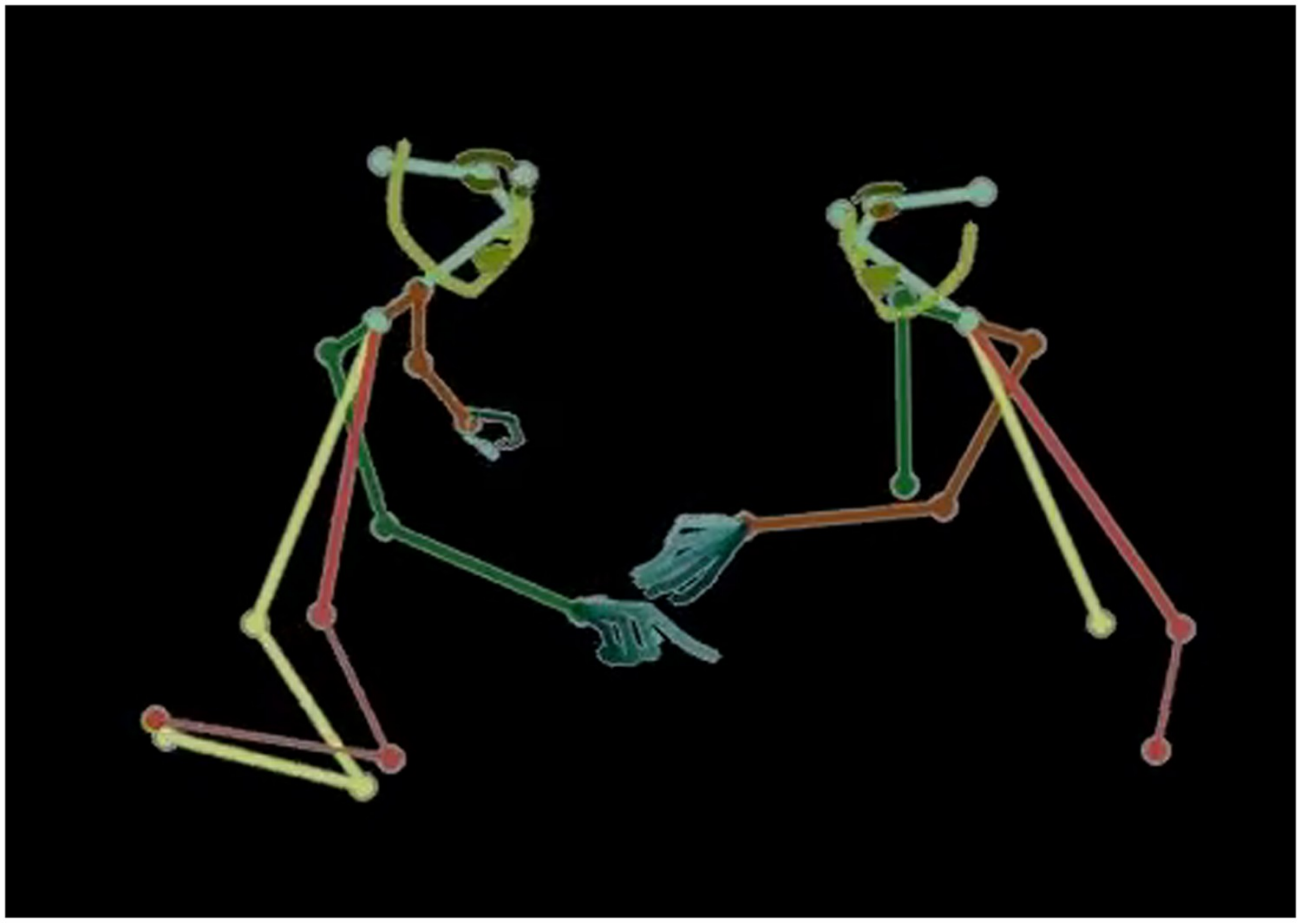
2D skeletons, including facial landmarks and hand details are automatically extracted using the OpenPose library [18].

**Fig 7:**
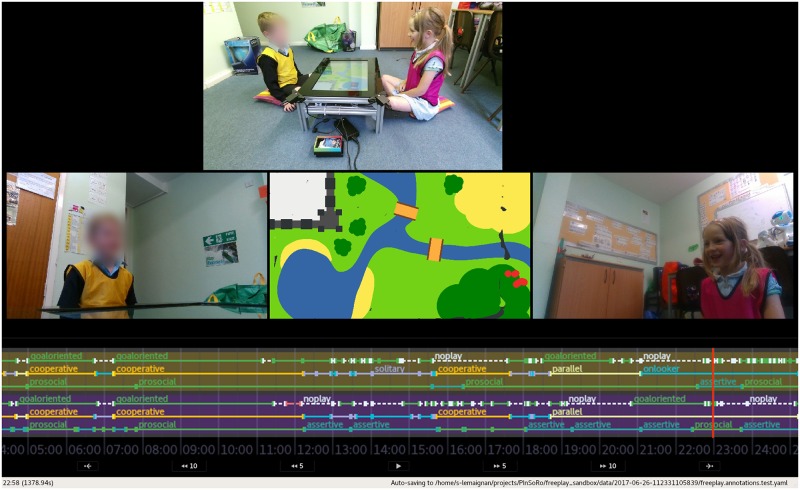
Screenshot of the dedicated tool developed for rapid annotation of the social interactions. The annotators used a secondary screen (tablet) with buttons (layout similar to Fig 5) to record the social constructs. Figure edited for legibility (timeline enlarged) and to mask out one of the children’ face. The right individual pictured in this figure has given written informed consent (as outlined in PLOS consent form) to appear.

**Fig 8:**
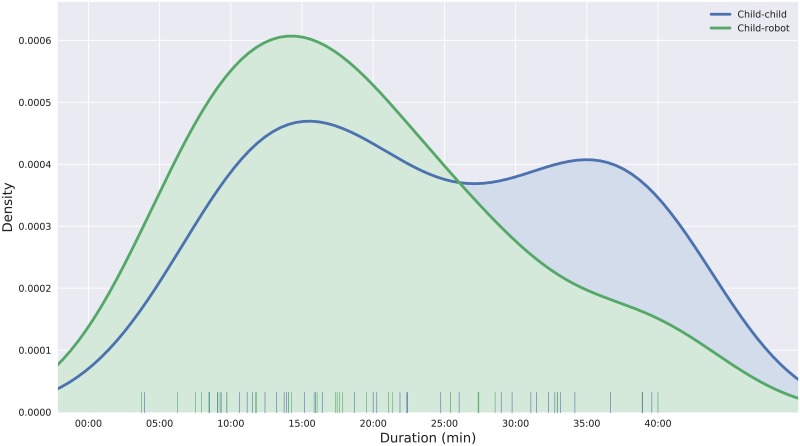
Density distribution of the durations of the interactions for the two conditions. Interactions in the child-robot condition were generally shorter than the child-child interactions. Interactions in the child-child condition followed a bi-modal distribution, with one mode centered around minute 15 (similar to the child-robot one) and one, much longer mode, at minute 37.

**Fig 9:**
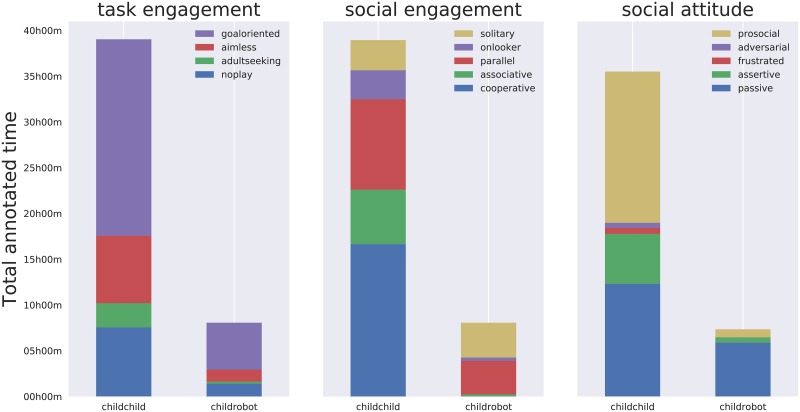
Repartition of annotations over the dataset (in total duration of recordings annotated with a given construct). The three classes of constructs (task engagement, social engagement, social attitude) and the two conditions (child-child and child-robot) are plotted separately.

**Fig 10:**
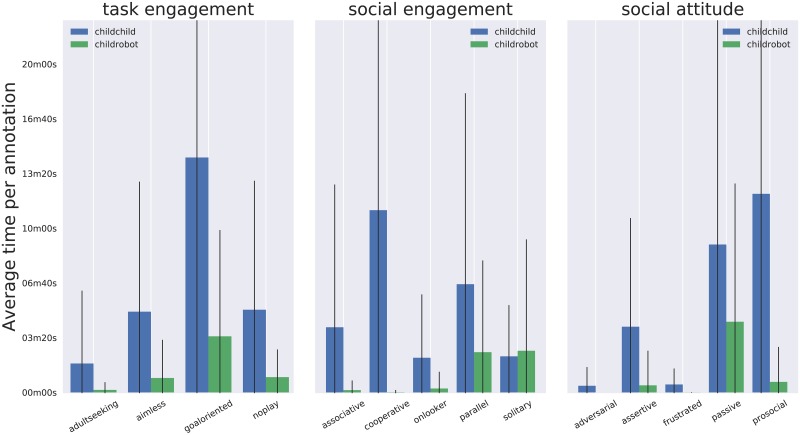
Mean time (and standard deviation) that each construct has been annotated in each recording. The large standard deviations reflect the broad range of group dynamics captured in the dataset.

**Fig 11:**
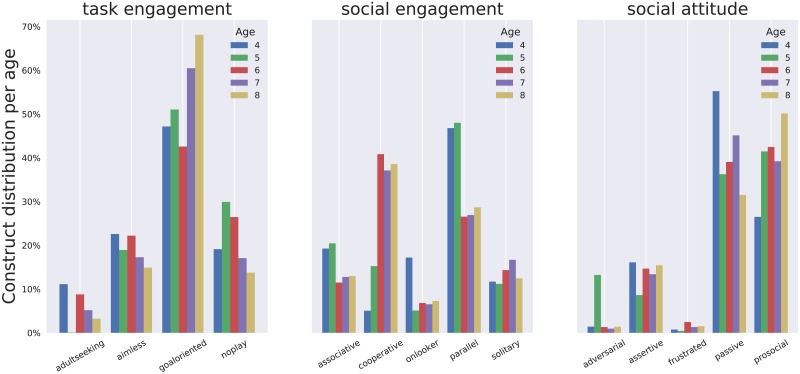
Percentage of observations for each constructs with respect the children’s age.

In the Wizard-of-Oz mode of operation, the experimenter would remotely control the robot through a tablet application developed for this purpose (Figs 3–11). The tablet exactly mirrored the game state, and the experimenter dragged the game items on the tablet as would the child on the touchscreen. On release, the robot would again mimic the dragging motion on the touchscreen, moving an object to a new location. This mode of operation, while useful to conduct controlled studies, was not used for the dataset acquisition.

#### Experiment manager

We developed as well a dedicated web-based interface (usually accessed from a tablet) for the experimenter to manage the whole experiment and data acquisition procedure (Figs 3–10). This interface ensured that all the required software modules were running; it allowed the experimenter to check the status of each of them and, if needed, to start/stop/restart any of them. It also helped managing the data collection campaign by providing a convenient interface to record the participants’ demographics, resetting the game interface after each session, and automatically enforcing the acquisition protocol (presented in Table 1).

**Table 1:** Data acquisition protocol.

**Greetings** ***(about 5 min)*** explain the purpose of the study: showing robots how children play briefly present a Nao robot: the robot stands up, gives a short message (*Today I’ll be watching you playing* in the child-child condition;*Today I’ll be playing with you* in the child-robot condition), and sits down. place children on cushions complete demographics on the tablet remind the children that they can withdraw at anytime
**Gaze tracking task** ***(40 sec)*** children are instructed to closely watch a small picture of a rocket that moves randomly on the screen. Recorded data is used to train a eye-tracker post-hoc.
**Tutorial** ***(1-2 min)*** explain how to interact with the game, ensure the children are confident with the manipulation/drawing.
**Free-play task (up to 40 min)** initial prompt: *“Just to remind you, you can use the animals or draw. Whatever you like. If you run out of ideas, there’s also an ideas box. For example, the first one is a zoo. You could draw a zoo or tell a story. When you get bored or don’t want to play anymore, just let me know.”* let children play once they wish to stop, stop recording
**Debriefing** ***(about 2 min)*** answer possible questions from the children give small reward (e.g. stickers) as a thank you

### Coding of the social interactions

Our aim is to provide insights on the social dynamics, and as such we annotated the dataset using a combination of three coding schemes for social interactions that reuse and adapt established social scales. Our resulting coding scheme (Fig 5) looked specifically at three axis: the level of *task engagement* (that distinguishes between *focused*, *task oriented* behaviours, and *disengaged*—yet sometimes highly social – behaviours); the level of social engagement (reusing Parten’s stages of play, but at a fine temporal granularity); the social attitude (that encoded attitudes like *supportive*, *aggressive*, *dominant*, *annoyed*, etc).

#### Task engagement

The first axis of our coding scheme aimed at making a broad distinction between ‘on-task’ behaviours (even though the free-play sandbox did not explicitly require the children to perform a specific task, they were still engaged in an underlying task: to play with the game) and ‘off-task’ behaviours. We called ‘on-task’ behaviours *goal oriented*: they encompassed considered, planned actions (that might be social or not). *Aimless* behaviours (with respect to the task) encompassed opposite behaviours: being silly, chatting about unrelated matters, having a good laugh, etc. These *Aimless* behaviours were in fact often highly social, and played an important role in establishing trust and cooperation between the peers. In that sense, we considered them as as important as on-task behaviours.

#### Social engagement: Parten’s stages of play at micro-level

In our scheme, we characterised *Social engagement* by building upon Parten’s stages of play [3]. These five stages of play are normally used to characterise rather long sequences (at least several minutes) of social interactions. In our coding scheme, we applied them at the level of each of the micro-sequences of the interactions: one child is drawing and the other is observing was labelled as *solitary play* for the former child, *on-looker* behaviour for the later; the two children discuss what to do next: this sequence was annotated as a *cooperative* behaviour; etc.

We chose this fine-grained coding of social engagement to enable proper analyses of the internal dynamics of a long sequence of social interaction.

#### Social attitude

The constructs related to the social *attitude* of the children derived from the *Social Communication Coding System* (SCCS) proposed by Olswang et al. [13]. The SCCS consists in 6 mutually exclusive constructs characterising social communication (*hostile*; *pro-social*; *assertive*; *passive*; *adult seeking*; *irrelevant*) and were specifically created to characterise children’s communication in a classroom setting.

We transposed these constructs from the communication domain to the general behavioural domain, keeping the *pro-social*, *hostile* (whose scope we broadened in *adversarial*), *assertive* (i.e. dominant), and *passive* constructs. In our scheme, the *adult seeking* and *irrelevant* constructs belong to Task Engagement axis.

Finally, we added the construct *Frustrated* to describe children who are reluctant or refuse to engage in a specific phase of interaction because of a perceived lack of fairness or attention from their peer, or because they fail at achieving a particular task (like a drawing).

### Protocol

We adhered to the acquisition protocol described in Table 1 with all participants. To ease later identification, each child was also given a different and brightly coloured sports bib to wear.

Importantly, during the *Greetings* stage, we showed the robot both moving and speaking (for instance, “Hello, I’m Nao. Today I’ll be playing with you. Exciting!” while waving at the children). This was of particular importance in the child-robot condition, as it set the children’s expectations in term of the capabilities of the robot: the robot could in principle speak, move, and even behave in a social way.

Also, the game interface of the free-play sandbox offered a tutorial mode, used to ensure the children know how to manipulate items on a touchscreen and draw. In our experience, this never was an issue for children.

### Data collection

Table 2 lists the raw datastreams that were collected during the game. By relying on ROS for the data acquisition (and in particular the rosbag tool), we ensured all the datastreams were synchronised, timestamped, and, where appropriate, came with calibration information (for the cameras mainly). For the PInSoRo dataset, cameras were configured to stream in qHD resolution (960×540 pixels) in an attempt to balance high enough resolution with tractable file size. It resulted in bag files weighting ≈1GB per minute.

**Table 2:** List of raw datastreams available in the PInSoRo dataset. Each datastream is timestamped with a synchronised clock to facilitate later analysis.

Domain	Type	Details
child 1	audio	16kHz, mono, semi-directional
	face (RGB)	qHD (960×540), 30Hz
	face (depth)	VGA (640×480), 30Hz
child 2	audio	16kHz, mono, semi-directional
	face (RGB)	qHD (960×540), 30Hz
	face (depth)	VGA (640×480), 30Hz
environment	RGB	qHD (960×540), 29.7Hz
game interactions	background drawing (RGB)	4Hz
	finger touches	6 points multi-touch, 10Hz
	game items pose	TF frames, 10Hz
other	static transforms between touchscreen and facial cameras	
	cameras calibration informations	

Besides audio and video streams, user interactions with the game were monitored and recorded as well. The background drawings produced by the children were recorded. They were also segmented according to their colours, and the contours of resulting regions were extracted and recorded. The positions of all manipulable game items were recorded (as ROS TF frames), as well as every touch on the touchscreen.

### Data post-processing

Table 3 summarises the post-processed datastreams that are made available alongside the raw datastreams.

**Table 3:** List of post-processed datastreams available in the PInSoRo dataset. With the exception of social annotations, all the data was automatically computed from the raw datastreams at 30Hz.

Domain	Type	Details
children	face	70 facial landmarks (2D)
		17 facial action-units
		head pose estimation (TF frame)
		gaze estimation (TF frame)
	skeleton	18 points body pose (2D)
		20 points hand tracking (2D, only when visible)
	audio	INTERSPEECH’s 16 low-level descriptors
annotations	timestamped annotations of social behaviours and remarkable events	

#### Audio processing

Audio features were automatically extracted using the OpenSMILE toolkit [14]. We used a 33ms-wide time windows in order to match the cameras FPS. We extracted the INTERSPEECH 2009 Emotion Challenge standardised features [15]. These are a range of prosodic, spectral and voice quality features that are arguably the most common features we might want to use for emotion recognition [16]. For a full list, please see [15]. As no reliable speech recognition engine for children voice could be found [17], audio recordings were not automatically transcribed.

#### Facial landmarks, action-units, skeletons, gaze

Offline post-processing was performed on the images obtained from the cameras. We relied on the CMU OpenPose library [18] to extract for each child the upper-body skeleton (18 points), 70 facial landmarks including the pupil position, as well as the hands’ skeleton (Fig 6).

This skeletal information was extracted from the RGB streams of each of the three cameras, for every frame. It is stored alongside the main data in an easy-to-parse JSON file.

For each frame, 17 action units, with accompanying confidence levels, were also extracted using the OpenFace library [19]. The action-units recognised by OpenFace and provided alongside the data are AU01, AU02, AU04, AU05, AU06, AU07, AU09, AU10, AU12, AU14, AU15, AU17, AU20, AU23, AU25, AU26, AU28 and AU45 (classification following https://www.cs.cmu.edu/~face/facs.htm).

Gaze was also estimated, using two techniques. First, head pose estimation was performed following [20], and used to estimate gaze pose. While this technique is effective to segment pose at a coarse level (i.e. gaze on interactive table vs. gaze on other child/robot vs. gaze on experimenter), it offers limited accuracy when tracking the precise gaze location on the surface of the interactive table (due to not tracking the eye pupils).

We complemented head pose estimation with a neural network (a simple 7-layers, fully connected, multi-layer perceptron with ReLU activations and 64 units per layer), implemented with the Caffe framework (source available here: https://github.com/severin-lemaignan/visual_tracking_caffe).

The network trained from a ground truth mapping between the children’ faces and 2D gaze coordinates. Training data is obtained by asking the children to follow a target on the screen for a short period of time before starting the main free play activity (see protocol, Table 1). The position of the target provides the ground truth (x, y) coordinates of the gaze on the screen. For each frame, the network is then fed a feature vector comprising 32 facial and skeletal (x, y) points of interest relevant to gaze estimation (namely, the 2D location of the pupils, eye contours, eyebrows, nose, neck, shoulders and ears). The training dataset comprises 80% of the fully randomized dataset (123711 frames) and the testing dataset the remaining 20% (30927 frames). Using this technique, we measured a gaze location error of 12.8% on our test data between the ground truth location of the target on the screen and the estimated gaze location (i.e. ±9cm over the 70cm-wide touchscreen). The same pre-trained network is then used to provide gaze estimation during the remainder of the free play activity.

#### Video coding

The coding was performed post-hoc with the help of a dedicated annotation tool (Fig 7) which is part of the free-play sandbox toolbox. This tool can replay and randomly seek in the three video streams, synchronised with the recorded state of the game (including the drawings as they were created). An interactive timeline displaying the annotations is also displayed.

The annotation tool offers a remote interface for the annotator (made of large buttons, and visually similar to Fig 5) that is typically displayed on a tablet and allow the simultaneous coding of the behaviours of the two children. Usual video coding practices (double-coding of a portion of the dataset and calculation of an inter-judge agreement score) were followed.

## Results—The PInSoRo dataset

Using the free-play sandbox methodology, we have acquired a large dataset of social interactions between either pairs of children or one child and one robot. The data collection took place over a period of 3 months during Spring 2017.

In total, 120 children were recorded for a total duration of 45 hours and 48 minutes of data collection. These 120 children (see demographics in Table 4; sample drawn from local schools) were randomly assigned to one of two conditions: the child-child condition (90 children, 45 pairs) and a child-robot condition (30 children). The sample sizes were balanced in favour of the child-child condition as the social dynamics that we ultimately want to capture are much richer in this condition.

**Table 4:** Descriptive statistics for the children.

Condition	Age Mean	Age SD	# girls	# boys
Whole group	6.4	1.3	55	65
Child-child	6.3	1.4	42	48
Child-robot	6.9	0.9	12	18

In both conditions, and after a short tutorial, the children were simply invited to freely play with the sandbox, for as long as they wished (with a cap at 40 min; cf. protocol in Table 1).

In the child-child condition, 45 free-play interactions (i.e. 90 children) were recorded with a mean duration M = 24.15 min (standard deviation SD = 11.25 min). In the child-robot condition, 30 children were recorded, M = 19.18 min (SD = 10 min).

Fig 8 presents the density distributions of the durations of the interactions for the two baselines. The distributions show that (1) the vast majority of children engaged easily and for non-trivial amounts of time with the task; (2) the task led to a wide range of levels of commitment, which is desirable: it supports the claim that the free-play sandbox is an effective paradigm to observe a range of different social behaviours; (3) many long interactions (>30 min) were observed, which is especially desirable to study social dynamics.

The distribution of the child-robot interaction durations shows that these interactions are generally shorter. This was expected as the robot’s asocial behaviour was designed to be less engaging. Often, the child and the robot were found to be playing side-by-side—in some case for rather long periods of time—without interacting at all (solitary play).

Over the whole dataset, the children faces were detected on 98% of the images, which validates the positioning of the camera with respect to the children to record facial features.

### Annotations

Five expert annotators performed the dataset annotation. Each annotator received one hour of training by the experimenters, and were compensated for their work.

In total, 13289 annotations of social dynamics were produced, resulting in an average of 149 annotations per record (SD = 136), which equates to an average of 4.2 annotations/min (SD = 2.1), and an average duration of annotated episodes of 48.8 sec (SD = 33.3). Fig 9 shows the repartition of the annotation corpus over the different constructs presented in Fig 5. Fig 10 shows the mean annotation time and standard deviation per recording for each construct.

Overall, 23% of the dataset was double-coded. Inter-coder agreement was found to be 51.8% (SD = 16.8) for task engagement annotations; 46.1% (SD = 24.2) for social engagement; 56.6% (SD = 22.9) for social attitude.

These values are relatively low (only partial agreement amongst coders). This was expected, as annotating social interactions beyond surface behaviours is indeed generally difficult. The observable, objective behaviours are typically the result of a superposition of the complex and non-observable underlying cognitive and emotional states. As such, these deeper socio-cognitive states can only be indirectly observed, and their labelling is typically error prone.

However, this is not anticipated to be a major issue for data-driven analyses, as machine learning algorithms are typically trained to estimate probability distributions. As such, divergences in human interpretations of a given social episode will simply be reflected in the probability distribution of the learnt model.

When looking at social behaviours with respect to age groups, expected behavioural trends are observed (Fig 11): *adult seeking* goes down when children get older; more *cooperative* play is observed with older children, while more *parallel* play takes place with younger ones. In constrast, the social attitudes appear evenly distributed amongst age groups.

### Dataset availability and data protection

All data has been collected by researchers at the University of Plymouth, under a protocol approved by the university ethics committee. The parents of the participants explicitly consented in writing to sharing of their child’s video and audio with the research community. The data does not contain any identifying information, except the participant’s images. The child’s age and gender are also available. The parents of the children in this manuscript have given written informed consent (as outlined in PLOS consent form) to publish these case details.

The dataset is freely available to any interested researcher. Due to ethical and data protection regulations, the dataset is however made available in two forms: a public, Creative Commons licensed, version that does not include any video material of the children (no video nor audio streams), and hosted on the Zenodo open-data platform: https://zenodo.org/record/1043508. The complete version that includes all video streams is freely available as well, but interested researchers must first fill a data protection form. The detail of the procedure are available online: https://freeplay-sandbox.github.io/application.

## Discussion of the free-play sandbox

The free-play sandbox elicits a loosely structured form of play: the actual play situations are not known beforehand and might change several times during the interaction; the game actions, even though based on one primary interaction modality (touches on the interactive table), are varied and unlimited (especially when considering the drawings); the social interactions between participants are multi-modal (speech, body postures, gestures, facial expressions, etc.) and unconstrained. This loose structure creates a fecund environment for children to express a range of complex, dynamics, natural social behaviours that are not tied to an overly constructed social situation. The diversity of the social behaviours that we have been able to capture can indeed been seen in Figs 9 and 11.

Yet, the interaction is nonetheless structured. First, the physical bounds of the interactive table limit the play area to a well defined and relatively small area. As a consequence, children are mostly static (they are sitting in front of the table) and their primary form of physical interaction is based on 2D manipulations on a screen.

Second, the game items themselves (visible in Fig 2) structure the game scenarios. They are iconic characters (animals or children) with strong semantics associated to them (such as ‘crocodiles like water and eat children’). The game background, with its recognizable zones, also elicit a particular type of games (like building a zoo or pretending to explore the savannah).

These elements of structure (along with other, like the children demographics) arguably limit how general the PInSoRo dataset is. However, it also enable the free-play sandbox paradigm to retain key properties that makes it a practical and effective scientific tool: because the game builds on simple and universal play mechanics (drawings, pretend play with characters), the paradigm is essentially cross-cultural; because the sandbox is physically bounded and relatively small, it can be easily transported and practically deployed in a range of environments (schools, exhibitions, etc.); because the whole apparatus is well defined and relatively easy to duplicate (it essentially consists in one single touchscreen computer), the free-play sandbox facilitates the replication of studies while preserving ecological validity.

Compared to existing datasets of social interactions (the *Multimodal Dyadic Behavior Dataset*, the *Tower Game* dataset and the *UE-HRI* dataset), PInSoRo is much larger, with more than 45 hours of data, compared to 10.6, 5.6 and 6.9 hours respectively. PInSoRo is fully multi-modal whereas the *Tower Game* dataset does not include verbal interactions, and the *UE-HRI* dataset focuses instead of spoken interactions. Compared to the *Multimodal Dyadic Behavior Dataset*, PInSoRo captures a broader range of social situations, with fully calibrated datastreams, enabling a broad range of automated data processing and machine learning applications. Finally, PInSoRo is also unique for being the first (open) dataset capturing *long sequences* (up to 40 minutes) of *ecologically valid* social interactions amongst children or between children and robots.

## Conclusion—Towards the machine learning of social interactions?

We presented in this article the PInSoRo dataset, a large and open dataset of loosely constrained social interactions between children and robots. By relying on prolonged free-play episodes, we captured a rich set of naturally-occurring social interactions taking place between pairs of children or pairs of children and robots. We recorded an extensive set of calibrated and synchronised multimodal datastreams which can be used to mine and analyse the social behaviours of children. As such, this data provides a novel playground for the data-driven investigation and modelling of the social and developmental psychology of children.

The PInSoRo dataset also holds considerable promise for the automatic training of models of social behaviours, including implicit social dynamics (like rhythmic coupling, turn-taking), social attitudes, or engagement interpretation. As such, we foresee that the dataset might play an instrumental role in enabling artificial systems (and in particular, social robots) to recognise, interpret, and possibly, generate, socially congruent signals and behaviours whenever interacting with children. Whether such models can help uncover some of the implicit precursors of social behaviours, and is so, whether the same models, learnt from children data, can as well be used to interpret adult social behaviours, are open—and stimulating—questions that this dataset might contribute to answer.
